# Supplementary material for: hu.MAP3.0: atlas of human protein complexes by integration of >25,000 proteomic experiments
Source: Mol Syst Biol. 2025 May 27;21(7):911–43. doi: 10.1038/s44320-025-00121-5 (PMC12222714; doi:10.1038/s44320-025-00121-5)
Supplement: Supplementary file 1 — Appendix [file 44320_2025_121_MOESM1_ESM.pdf]

Appendix for ***"hu.MAP3.0: Atlas of human protein complexes by integration of > 25,000 proteomic experiments."***

<u>**Table of Contents**</u>

Appendix Figure S1. Median complex covariation does not strongly correlate with hu.MAP3.0 confidence levels. **(Page 2)**
Appendix Figure S2. Complex abundance levels vary between cancer lineages. **(Page 2)**
Appendix Figure S3. Statistical enrichment of hu.MAP3.0 complex abundance across cancer lineages. **(Page 3)**
Appendix Figure S4. Proteins from high probability negative pairs have more similar Gene Ontology (GO) terms and random pairs. **(Page 4)**
Appendix Figure S5. Precision-Recall plot evaluated on the leave-out test set of gold standard interactions for different versions of hu.MAP3.0. **(Page 4)**

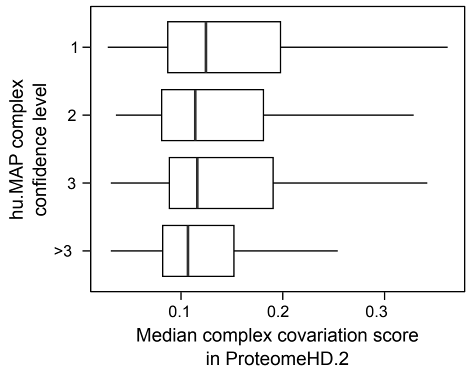

Appendix Figure S1. Median complex covariation does not strongly correlate with hu.MAP3.0 confidence levels. The median of protein pairwise covariation probability from ProteomeHD.2 for subunits belonging to the same hu.MAP3.0 complex was calculated, for complexes that had more than 50% coverage in ProteomeHD.2. Box corresponds to quartiles of the distribution, line in box corresponds to median, whiskers extend to the first data point inside the first/third quartile plus 1.5 × interquartile range (IQR), respectively.

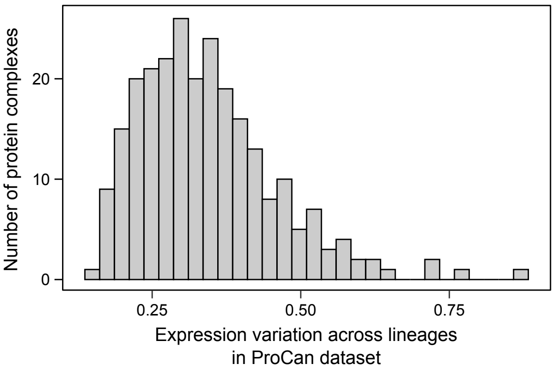

Appendix Figure S2. Complex abundance levels vary between cancer lineages. Relative protein expression across cancer lineages was averaged to hu.MAP3.0 complex-level to identify complexes that differ in abundance between lineages, as quantified by the standard deviation of the complex-level z-score. Subunits with more than 80% missing values in the ProCAN dataset were excluded from downstream analysis.

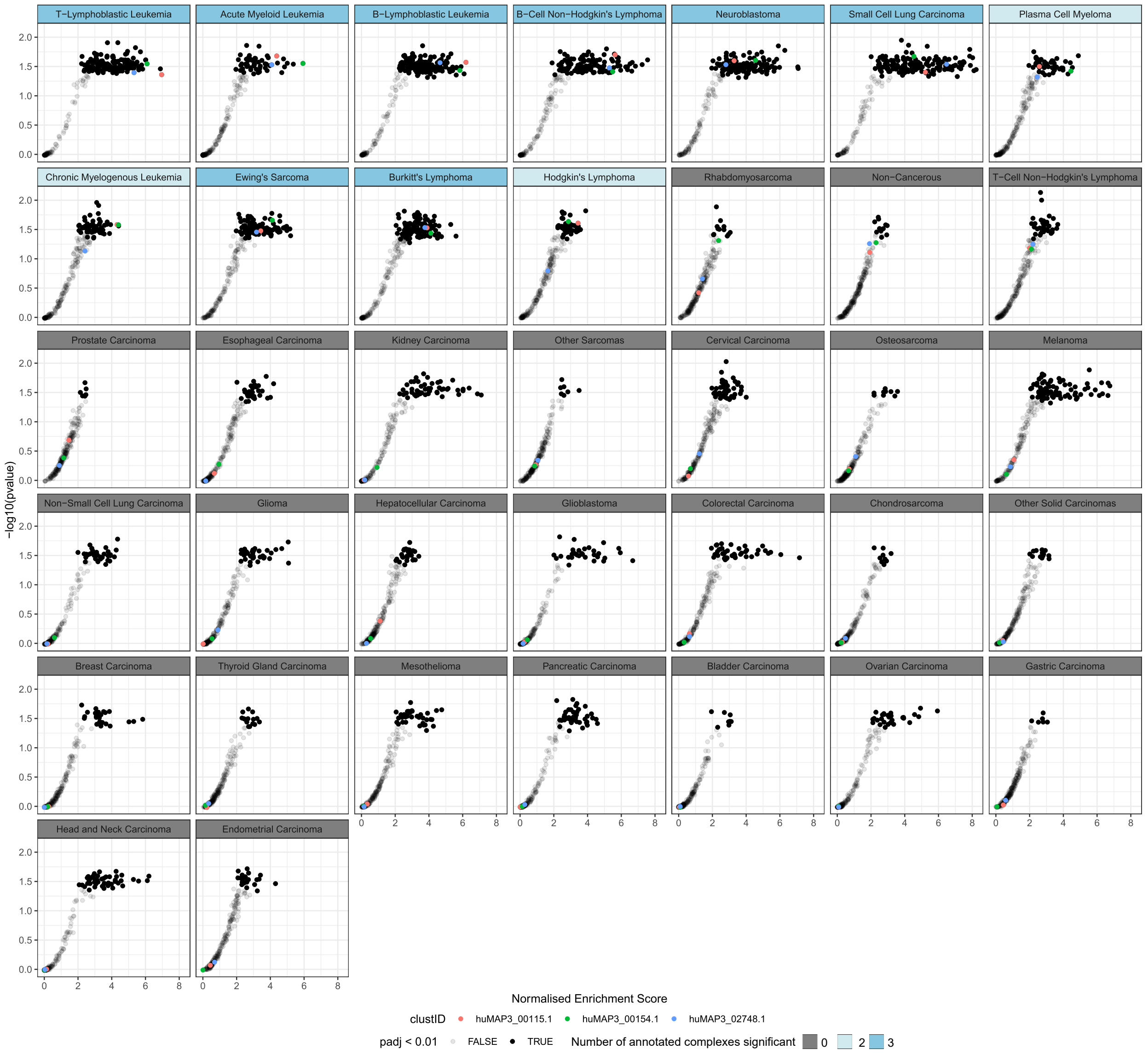

Appendix Figure S3. Statistical enrichment of hu.MAP3.0 complex abundance across cancer lineages. An overrepresentation test was performed to identify complexes that are overexpressed in certain cancer lineages. Each point represents a hu.MAP3.0 complex, with its relative lineage-enrichment score on the x-axis and the overrepresentation significance on the y-axis. Complexes with FDR-adjusted p-values below 0.01 are shown in black and selected complexes are annotated. Lineages with significant overexpression of all three of these selected complexes are shown in dark blue, and those with two complexes reaching significance in light blue. Protein subunits with more than 80% missing values in the cancer lineage dataset were excluded from this analysis.

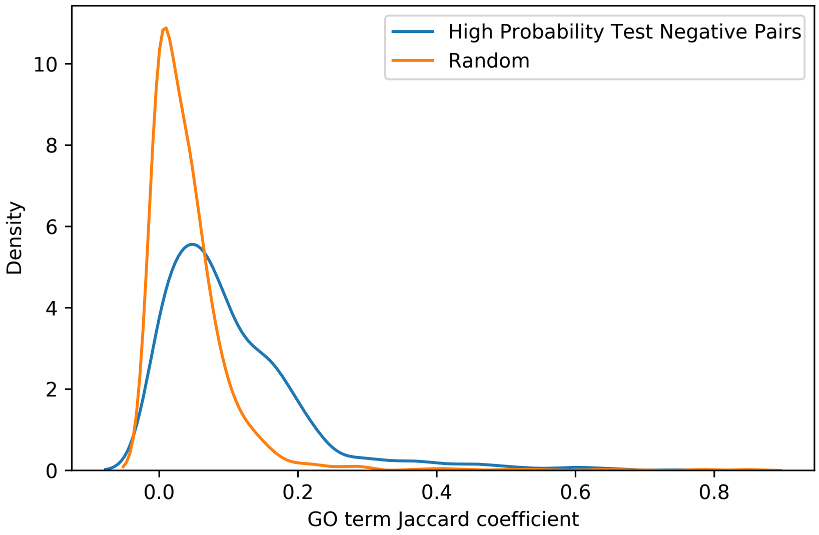

Appendix Figure S4. Proteins from high probability negative pairs have more similar Gene Ontology (GO) terms and random pairs. Orange distribution shows random pairs GO term overlap. Blue distribution shows GO term overlap of negative pairs from test set with high probability (huMAP3.0 confidence score > 0.9).

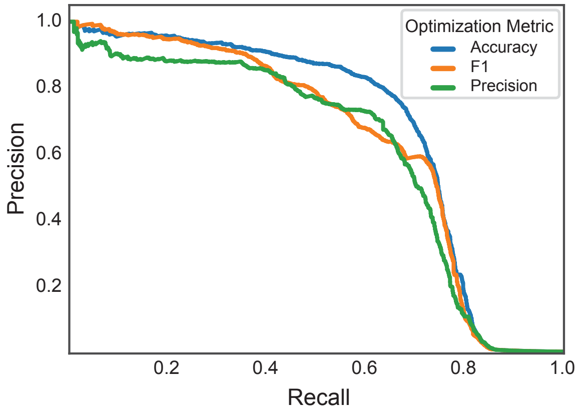

Appendix Figure S5. Precision-Recall plot evaluated on the leave-out test set of gold standard interactions for different versions of hu.MAP3.0. The different models were trained with the same literature-curated training set but optimized for different evaluation metrics. The accuracy optimized model shows the greatest performance and is designated as the hu.MAP3.0 model.
