# Supplementary material for: hu.MAP3.0: atlas of human protein complexes by integration of >25,000 proteomic experiments
Source: Mol Syst Biol. 2025 May 27;21(7):911–43. doi: 10.1038/s44320-025-00121-5 (PMC12222714; doi:10.1038/s44320-025-00121-5)
Supplement: Supplementary file 14 — Expanded View Figures [file 44320_2025_121_MOESM14_ESM.pdf]

Expanded View Figures

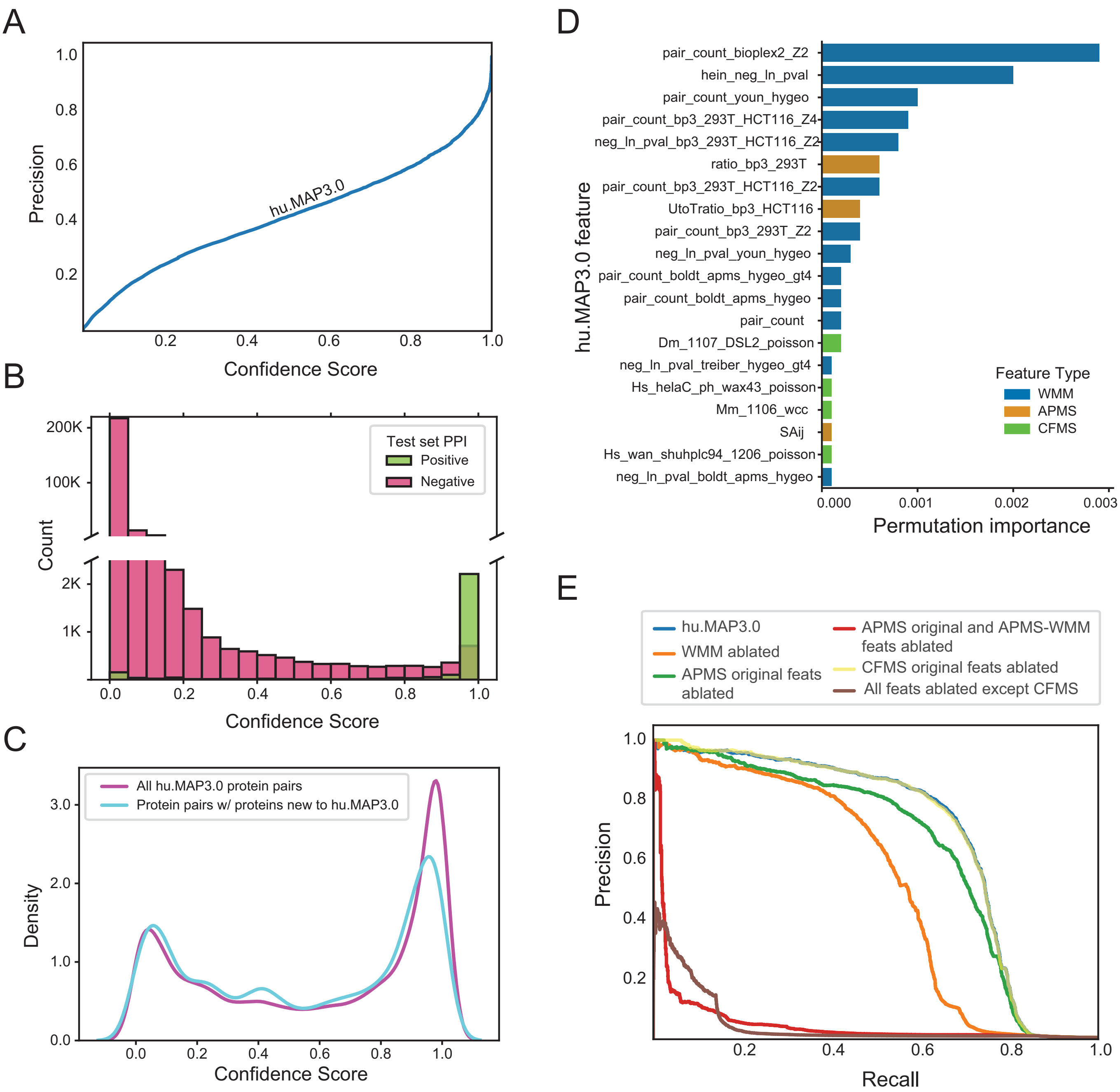

Figure EV1. Characterization of hu.MAP3.0 model performance.

(**A**) Line plot depicting the relationship between precision and the hu.MAP3.0 model confidence score on the test set interactions, illustrating that increases in confidence score correspond with increased precision. (**B**) Distribution of positive and negative test set interactions across model confidence scores. The histogram highlights the separation of these interactions. (**C**) Distribution of hu.MAP3.0 confidence scores for all pairs (violet) and pairs with protein new to hu.MAP3.0 (light blue). (**D**) Autogluon feature importance for top 20 evidence features used in hu.MAP3.0 machine learning classifier. Colors represent feature categories including Weighted Matrix Model (WMM), affinity purification based features (APMS), and co-fractionation (CFMS). (**E**) Precision recall curves for different feature category ablation tests.

© The Author(s)

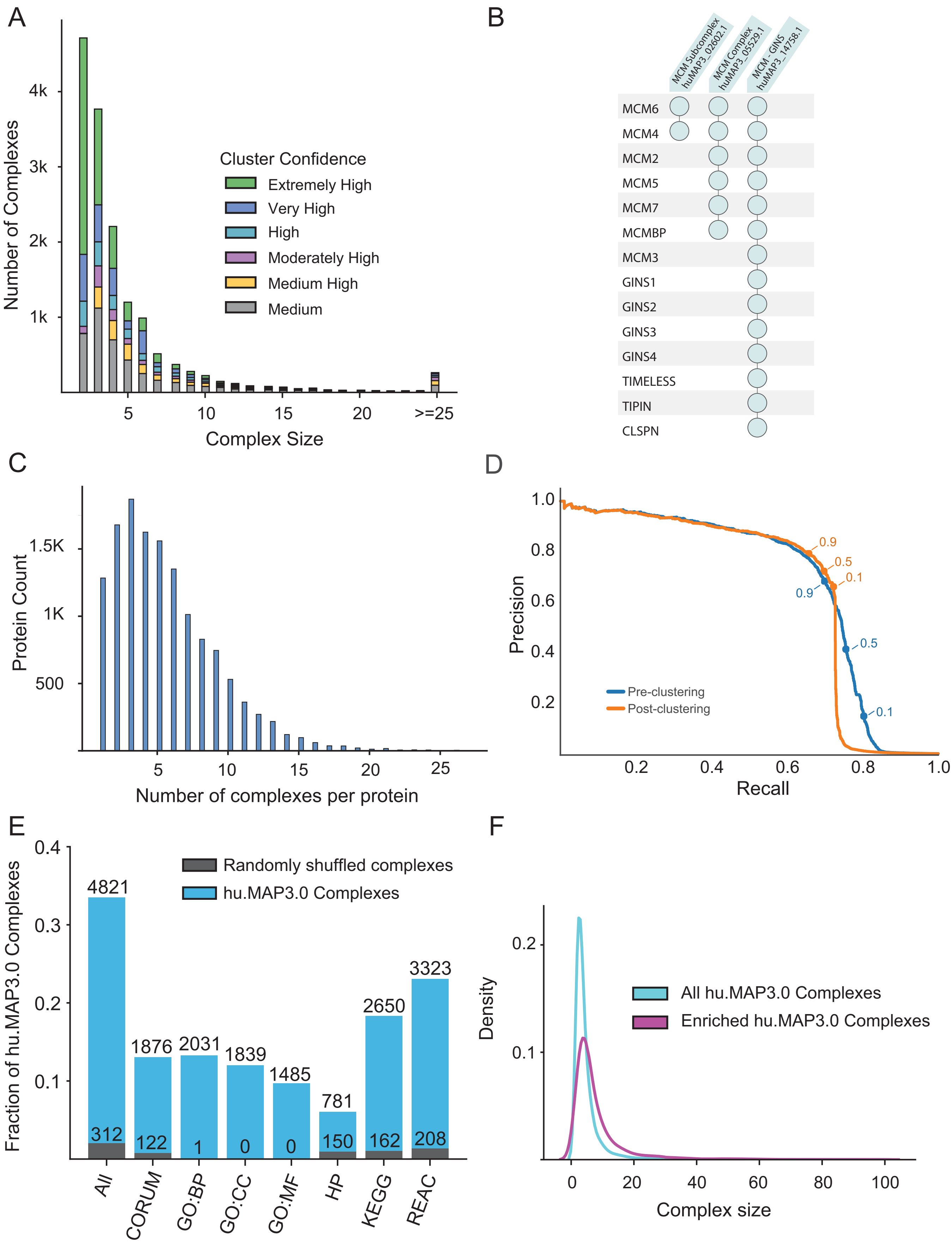
A
Number of Complexes
4k
3k
2k
1k
5
10
15
20
>=25
Complex Size
Cluster Confidence
Extremely High
Very High
High
Moderately High
Medium High
Medium
B
MCM Subcomplex huMAP3_02602.1
MCM Complex huMAP3_05529.1
MCM - GINS huMAP3_14758.1
MCM6
MCM4
MCM2
MCM5
MCM7
MCMBP
MCM3
GINS1
GINS2
GINS3
GINS4
TIMELESS
TIPIN
CLSPN
C
Protein Count
1.5K
1K
500
5
10
15
20
25
Number of complexes per protein
D
Precision
1.0
0.8
0.6
0.4
0.2
Recall
0.2
0.4
0.6
0.8
1.0
0.9
0.5
0.1
0.9
0.5
0.1
Pre-clustering
Post-clustering
E
Fraction of hu.MAP3.0 Complexes
0.4
0.3
0.2
0.1
Randomly shuffled complexes
hu.MAP3.0 Complexes
4821
312
1876
122
2031
1
1839
0
1485
0
781
150
2650
162
3323
208
All
CORUM
GO:BP
GO:CC
GO:MF
HP
KEGG
REAC
F
Density
0.2
0.1
0
20
40
60
80
100
Complex size
All hu.MAP3.0 Complexes
Enriched hu.MAP3.0 Complexes

 © The Author(s)

Figure EV2. Clustering size distribution and annotation enrichment.

(**A**) Distribution of hu.MAP3.0 complex size. Colors in stacked bars represent cluster confidence. (**B**) Subunit composition of MCM complex hierarchy. (**C**) Distribution of the number of complexes assigned to individual proteins. (**D**) Precision-Recall plot for the hu.MAP3.0 model on test set interactions pre- and post-clustering, with confidence score thresholds marked as circles, demonstrating clear separation along the curve. (**E**) Annotation enrichment of hu.MAP3.0 complexes using g:Profiler. Bars represent the number of complexes enriched for GO, KEGG, CORUM, Reactome, or Human Phenotype Ontology (HP) annotations that pass a corrected *p*-value of 0.01. Random represents enriched protein sets when complex membership is shuffled for all complexes. (**F**) Distribution of hu.MAP3.0 complex size for all complexes (light blue) and complexes enriched with annotation (violet).

© The Author(s)

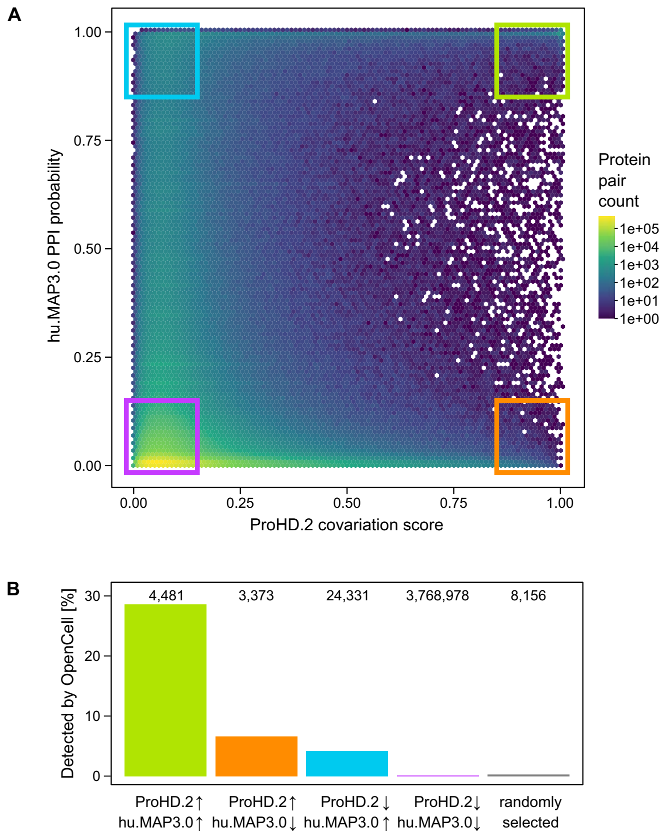

Figure EV3. Comparison of ProteomeHD.2, hu.MAP3.0, and OpenCell.

(**A**) Scatterplot of protein pairs' ProteomeHD.2 covariation score (x-axis) and hu.MAP3.0 confidence score (y-axis). Four quadrants are highlighted where yellow = high in hu.MAP3.0, high in ProteomeHD.2, blue = high in hu.MAP3.0, low in ProteomeHD.2, orange = low in hu.MAP3.0, high in ProteomeHD.2, and purple = low in both. (**B**) Comparison of four quadrants in (**A**) to an orthogonal protein interaction dataset, OpenCell, not included in either hu.MAP3.0 nor ProteomeHD.2. Bars represent the percent detected in OpenCell. Number of protein pairs per category are shown above the bars.

 © The Author(s)

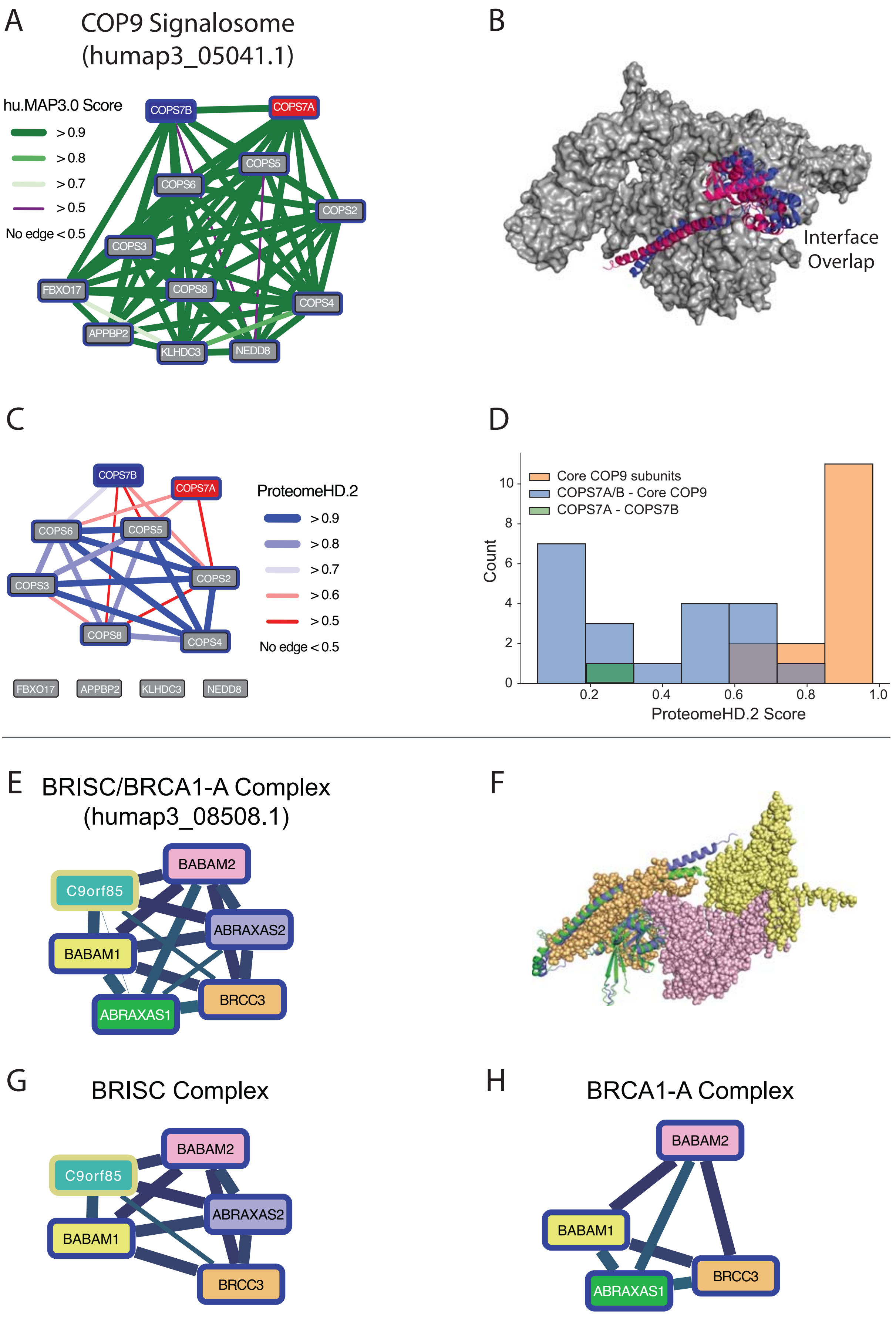
A
COP9 Signalosome (humap3_05041.1)
hu.MAP3.0 Score
> 0.9
> 0.8
> 0.7
> 0.5
No edge < 0.5
COPS7B
COPS7A
COPS5
COPS6
COPS2
COPS3
FBXO17
COPS8
COPS4
APPBP2
KLHDC3
NEDD8
B
Interface Overlap
C
ProteomeHD.2
> 0.9
> 0.8
> 0.7
> 0.6
> 0.5
No edge < 0.5
FBXO17
APPBP2
KLHDC3
NEDD8
D
Core COP9 subunits
COPS7A/B - Core COP9
COPS7A - COPS7B
Count
ProteomeHD.2 Score
E
BRISC/BRCA1-A Complex (humap3_08508.1)
BABAM2
C9orf85
ABRAXAS2
BABAM1
BRCC3
ABRAXAS1
F
G
BRISC Complex
H
BRCA1-A Complex

Figure EV4. Example complexes with mutual exclusive subunits.

(**A**) Co-complex interaction network of the COP9 Signalosome shows high interconnectivity of core subunits and peripheral subunits FBXO17, APPBP2, KLHDC3, and NEDD8. (**B**) Structure of COP9 Signalosome core subunits show overlapping interfaces of COPS7A and COPS7B with other core subunits (PDB id: 4D10, 6R6H) pointing to these subunits being mutually exclusive. (**C**) Protein covariation network of COP9 Signalosome from ProteomeHD.2. Core subunits of the network are highly co-expressed while COPS7A and COPS7B are less co-expressed to core subunits. (**D**) Distributions of covariation between core COP9 subunits (orange), COPS7A or COPS7B and core COP9 subunits (blue), and the covariation between COPS7A and COPS7B (green). (**E**) The BRISC/BRCA1-A Complex highlights the identification of two mutually exclusive proteins and the annotation of an uncharacterized protein. Blue border of protein subunits represents a known member of the complex; the yellow border represents an uncharacterized protein. Edge weights represent hu.MAP3.0 confidence score. (**F**) AlphaFold3 model of known subunits of complex. The interfaces of ABRAXAS1 and ABRAXAS2 with BRCC3 overlap and therefore are determined to be mutually exclusive subunits. (**G**, **H**) BRISC/BRCA1-A Complex split into two complexes which appropriately separates mutually exclusive subunits and places the uncharacterized protein, C9ORF85, with the BRISC complex.

 © The Author(s)

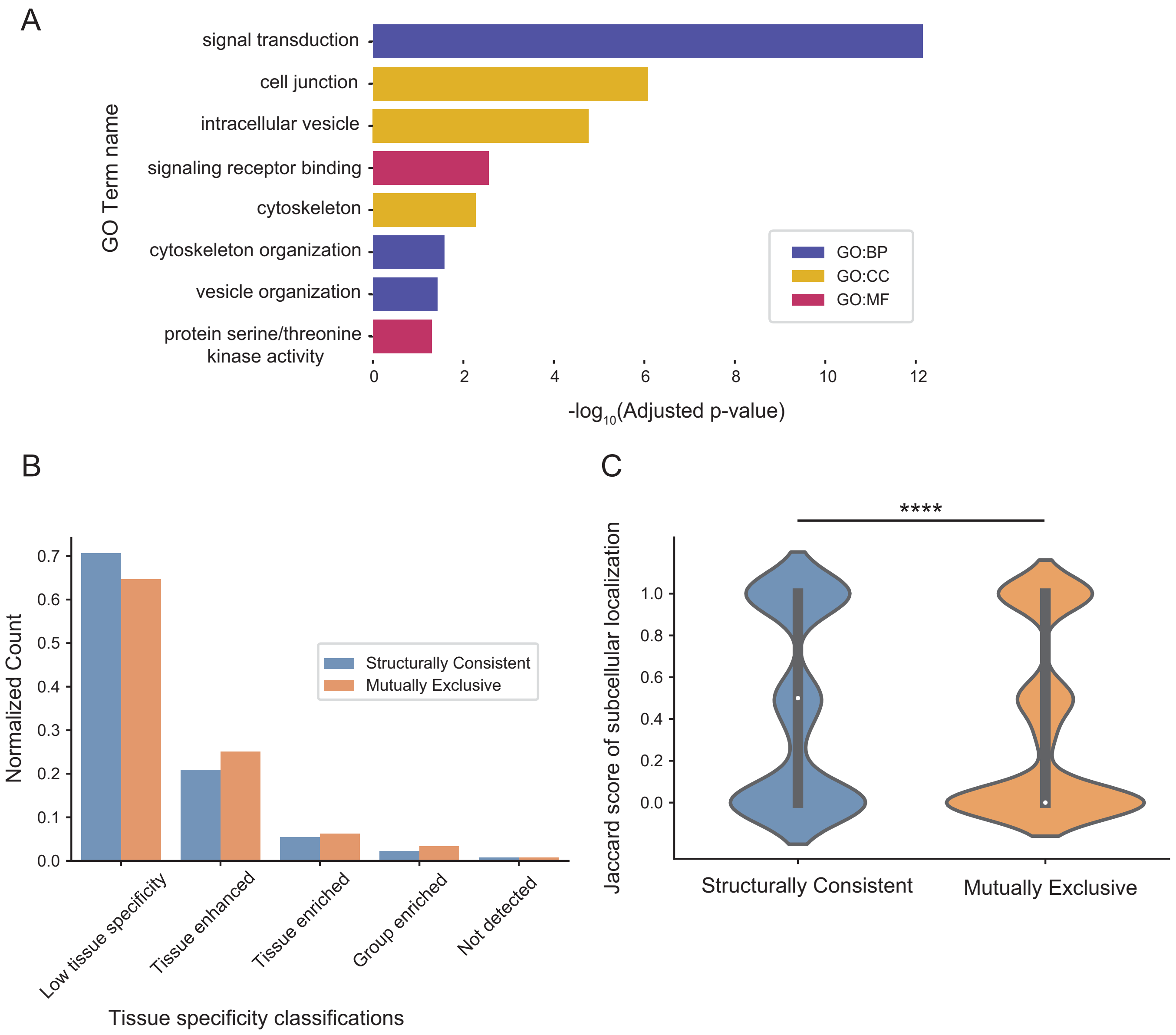

Figure EV5. Characterization of mutually exclusive interactions.

(**A**) Barplot displaying gene ontology annotation enrichments for molecular function (MF), biological process (BP), and cellular compartment (CC) for identified mutually exclusive proteins. (**B**) Barplot showing the normalized count of proteins from mutually exclusive or structurally consistent protein pairs in different Human Protein Atlas tissue expression classifications. (**C**) Violin plot showing the distribution of Jaccard similarity of subcellular localizations between proteins in mutually exclusive ($N = 4031$) and structurally consistent ($N = 1830$) protein pairs (two-sided Mann-Whitney *U*-Test, ****$P < 0.0001$, $P = 4.48 \times 10^{-13}$). Violin shape indicates density of data points, with wider sections indicating higher concentration of values. The upper and lower bounds of the inner box indicate the quartiles of the distribution with first (Q1, 25th percentile) and (Q3, 75th percentile), respectively. Here, the first and third quartile are the minimum and maximum values, respectively. The inner dot indicates the median.
